# Networks of exchange: East German kidney transplantation in European context, 1965–1990

**DOI:** 10.1017/mdh.2025.2

**Published:** 2025-10

**Authors:** Alexa Geisthövel

**Affiliations:** Charité Universitätsmedizin, Institute for the History of Medicine and Ethics in Medicine, Thielallee 71, 14195 Berlin, Germany

**Keywords:** Kidney transplantation, Organ exchange, Transnational collaboration, GDR, Cold War, Intertransplant

## Abstract

When kidney transplantation evolved from an experimental into a clinical treatment of end-stage renal disease (ESRD) in the 1960s, it was conceptualised as a collaborative therapy. Before specific immunosuppressants were introduced in the 1980s, the best chances for patient and graft survival were expected from finding ‘good’ matches between donor and recipient tissues. Therefore, the pioneers of clinical transplantation in Europe started to recombine their growing patient pools. They created trans-border organ exchange organisations such as Eurotransplant and Intertransplant, based on shared patient databases.

The article traces international and transnational co-operation in kidney exchange using the example of state-socialist Germany. How did the German Democratic Republic (GDR) get involved with the interconnected networks of knowledge, data, and organ exchange in ESRD treatment? In what ways did the domestic system of kidney transplantation depend on intra- and trans-bloc exchange? How did the GDR profit, and what did it have to offer on an international scale, both in the First and the Second World? The article sheds light on the under-explored transplantation history of the socialist East and thereby investigates the possibilities and limits of trans-bloc collaboration in Cold War Europe.

In 1973, the front page of a major East German newspaper reported that ‘urgent help had arrived by plane from Moscow’. Three weeks earlier, so the article read, the Berlin transplantation centre had received a donor kidney from the Soviet Institute for Experimental and Clinical Surgery and transplanted it to a 37-year-old worker suffering from end-stage renal disease (ESRD), who had recently left the hospital fully restored to health.^1^ If this incident of medical co-operation seems to have occurred within the expected realms of official German-Soviet Friendship, another press item from 1977 tells a more complex story: for the kidneys of a brain-dead person in New York with the rare blood group AB, no recipient could be found in the United States or through the West European Eurotransplant organisation. The well-connected East German transplantation surgeons had learned about this opportunity, and thanks to excellent Czech-German co-operation within the socialist Intertransplant organisation, one US kidney had finally been transplanted to a Czech citizen.^2^

These two samples of state-socialist journalism reflect the mixed messages that generally can be found in the coverage celebrating transplantation as an outstanding achievement. On the one hand, clinical kidney transplantation became a showcase for Cold War competition and the global comparison of health care systems. On the other hand, international collaboration created opportunities to be recognised as an advanced, peaceful society and to gather knowledge about the latest developments. As organ transplantation represented one of the most advanced areas of biomedicine, it signalled the GDR’s modernity and competitiveness to the world and promised East German citizens that health care would not only be universal and free but would also meet international standards.^3^

Still, the two articles cited above raise several questions with regard to the international dimension of this endeavour, especially if seen against the backdrop of the GDR’s relative isolation in the global political arena in the 1950s and 1960s. How did the GDR get involved and participate in the interconnected networks of knowledge, data and organ exchange in ESRD treatment? In what ways did the domestic system of kidney transplantation depend on intra- and trans-bloc exchange? Where did the GDR profit, and what did it have to offer on an international scale, both in the First and the Second World? And how did this complex, resource-intensive, specialised area of medicine resonate with the more general outlook of East German foreign policy?

My chapter will draw on current historiography, which puts the GDR in a transnational perspective. The obvious starting point has been inter-German histories, which showed that despite the marked ideological and political rivalry – including the West German policy of isolating the GDR internationally – many parallels, convergences, and entanglements can be observed in the field of medicine and health care.^4^ Recently, a new focus on the GDR’s relations to the Global South has emerged, often again in comparison with the Federal Republic of Germany (FRG).^5^ In contrast, health-related intra-socialist connections and interchanges, as exemplified by the Intertransplant project, have only just begun to be investigated. I can build on several studies that have followed trajectories of medical knowledge, people, and objects that multidimensionally connected the GDR to Western, Eastern, and Southern states, as well as to medical companies and international agencies, such as the World Health Organisation (WHO).^6^

As kidney transplantation has been historically defined by the interplay of ‘activities, participants, and structures at transnational, national, and local levels’,^7^ it offers an excellent opportunity to broaden our understanding of those complex interactions and their changes over time. On a national level, different medical disciplines and institutional actors had to interact in the creation and maintenance of a kidney transplantation system; apart from the kidney transplantation centres, this included decentralised infrastructure for chronic dialysis, immunological laboratories for tissue typing, committees for the determination of brain death, and surgical teams for organ procurement.^8^ In the 1960s, when clinical ESRD treatment was new and resources were limited everywhere, advances in any of these different fields mostly arrived through learning from abroad. Theoretical and practical knowledge was disseminated from a few pioneering centres around the globe, via person-to-person contacts, and through emerging cross-border professional communities, such as the International Society of Nephrology (ISN, 1960), the European Dialysis and Transplant Association (EDTA, 1963), and the International Histocompatibility Workshops (1964). I want to investigate how GDR physicians interacted with this unfolding international landscape and how their expectations and the stakes shifted during the 1970s and 1980s against the backdrop of general developments, such as the GDR’s international standing or the structural crisis of public debt.

My story relies on a specific accumulation of documents that characterised socialist statehood in East Germany. One main group of records originates from the Ministry of Health Care, which extensively monitored and controlled the activities of physicians and medical associations. The same goes for the documents of the Ministry of State Security. Several key players in dialysis and kidney transplantation were approached to report as unofficial informants to the secret police (and were likewise spied on by their colleagues). What we do not find are indications of illegal organ trafficking, neither with regard to East German citizens nor non-citizen residents or transit travellers between the German-German border and West Berlin. The absence of such documents does not mean that nothing illegal or unethical happened.^9^ However, the focus of my article will be on official, legal, and non-commercial kidney transplantation.

The first subchapter will introduce the key medical players who held major positions in ESRD treatment and travelled extensively. I will then outline how the immunological understanding of transplantation encouraged trans-border co-operation early on, followed by a sketch of how Intertransplant was established. East German kidney exchange activity will be analysed and placed in a wider context of exchange relations before conclusions are drawn for the overall questions of this special issue.

## ‘Three red musketeers’

Recent historiography has shown that international and transnational medical exchange in state-socialist countries very much depended on mobile individuals, oftentimes professionals and scientists who were travel cadres and were well-connected both in the domestic arena and with their peers abroad. Though committedly or opportunistically promoting the advancement of their home country and the ‘socialist camp’, they usually had a professional agenda of their own, which did not neatly coincide with the supposedly unified state interest or party line.^10^ This observation holds true for East German ESRD treatment as well. Its story can be told through the lens of some of those medical diplomats, dubbed the ‘three red musketeers’ by their Western colleagues when they entered the international arena in the 1960s.^11^

If you had to single out the one most important figure in East German kidney transplantation, it would be urologist Moritz Mebel (1923–2021). He led the team that performed the first successful East German kidney transplantation in 1967, kicking off the national kidney transplantation programme. In 1969/70, he was the founding director of the first kidney transplantation centre based at the Friedrichshain Municipal Hospital in Berlin, to be followed by centres in Halle (1974), Rostock (1976), and at the Charité University Hospital in Berlin (1983). With the Friedrichshain centre serving as headquarters for all of the GDR, Mebel was *de facto* chief co-ordinator of kidney transplantation, despite the fact that he was only officially appointed the Minister of Health Care’s Representative for Kidney Transplantation in 1983, after he had become head of the Charité Urological University Clinic.

However, as Mebel himself pointed out, it was nephrologists who first initiated international contact with colleagues in non-socialist countries.^12^ A pioneer in both respects was Harald Dutz (1914–2010), who started haemodialysis at the medical clinic of Rostock University in the late 1950s. Summoned back to the Charité in East Berlin to compensate for the exodus of many high-profile physicians, he headed the Charité’s Second Medical Clinic from 1962 until 1979 and helped to establish a nationwide network of dialysis centres. Back in Rostock, Horst Klinkmann (b. 1935) had started his clinical career under Dutz and contributed a marked interest in the technical aspects of dialysis. From 1974 until 1989, he was head of the medical clinic at Rostock University and enjoyed the leeway that the distance from the capital allowed. Due to his managerial qualities and international networks, he was appointed president of the Council for Medical Sciences in the GDR in 1982.^13^ In comparison with these three, the haematologists at the Berlin District Institute for Blood Donation and Transfusions, who managed the East German immunological reference laboratory, played only a secondary role.

Mebel, Dutz, and Klinkmann not only represented two medical subdisciplines and different aspects of ESRD treatment, but they also covered different geographical areas when it came to international co-operation. Mebel’s biography inclined him towards the Eastern bloc: born into a Jewish communist family with origins in Eastern Europe, his parents had left Germany in 1932/3 to raise their children in the Stalinist Soviet Union. In the 1940s, Mebel volunteered for the Red Army, studied medicine in Moscow, and qualified as a urologist before returning to Germany in 1958. He identified a hundred per cent with the German socialist state project.^14^ After his retirement, in 1986, he was elected to the Central Committee of the Socialist Unity Party. Due to his impeccable ideological record and political connections, he was in a position to pressure the health administration, though not always successfully. Fluent in Russian language and culture, he guaranteed access to decision makers in Moscow and ensured friendly relations with colleagues throughout Eastern Europe and the socialist Global South (without his network being restricted to socialist countries).

Harald Dutz, in contrast, was not a natural supporter of the GDR, but as a highly sought-after professional, he was flexible and loyal enough to earn himself a distinguished clinical career and influential positions. His correspondence testifies to diligent cross-border networking, supported by his closest international ally, Swedish pioneer of blood cleansing treatment Nils Alwall (1904–1986), who was on friendly terms with peers in both East and West and a key figure in international co-operation in ESRD treatment.^15^ Horst Klinkmann represented an even more profiled Western orientation. In 1969/70, he spent a year at Willem Kolff’s (1911–2009) clinic in Salt Lake City, a major hub for the development of artificial organs. Having returned to the GDR without hesitation, he capitalised on the language skills and connections he had acquired, both in the academic field and with Western producers of dialysis technology.

Each of these figures had their own agenda, but they managed to combine their efforts productively. In 1967, Dutz and Mebel convinced the Ministry of Health Care, as well as state and party leadership, to set up a state-sponsored nationwide dialysis programme, following the model of Sweden and the UK, which was a prerequisite for the transplantation programme.^16^ In the competition for financial, human, and technical resources in medicine, they successfully lobbied for a treatment that benefitted only a comparatively small number of patients. Likewise, Mebel, Dutz, and Klinkmann complemented their respective strengths when it came to fostering international ties.

## Collaboration as a (perceived) necessity: The HLA system, tissue typing, and the beginnings of organ exchange in Europe

In the 1960s, kidney transplantation evolved from an experimental into a clinical treatment of ESRD in the Global North. Chronic renal failure was the first fatal disease that became manageable through a combination of long-term dialysis and transplantation. Working together over spatial distances and exchanging between different health systems were considered necessities from the start, surpassing the usual level of international collaboration in medicine.^17^ The reason for this was the problem of tissue incompatibility that would make the immune system of the recipient reject the transplanted organ if tissue profiles differed too much. In the late 1950s, the first human leukocyte antigen (HLA) group was described. It was but a small sector of the vast and highly variable major histocompatibility complex, a series of genes located on the sixth chromosome. This anti[body-]gen[erating] molecular structure was found to bind antibodies during an acquired immune response.

The expansion of kidney transplantation as a regular clinical treatment meant that organ donation from living relatives with similar tissue profiles would not provide kidneys for every patient in need. With local deceased non-relatives, so-called cadaveric donors, on the other hand, tissue compatibility was unpredictable and improbable in most cases. Thus, in order to expand kidney transplantation, two approaches seemed possible. One was to suppress the natural immune response with radiation or pharmacologically. Effective immunosuppressive drugs had been in use since 1963, but the therapy was still unspecific and put the transplanted patient at a high risk of dying from a serious infection. It was not until the early 1980s that the new drug cyclosporine A would largely solve this problem. The other option was finding ‘good matches’ in the multitude of possible combinations between donor and recipient tissues. In particular, European transplantologists believed it necessary to ensure at least a certain degree of HLA compatibility, though the significance of the HLA-D region in this process was properly understood only in the 1970s.^18^

Before matching could be put into practice on a clinical scale, the HLA system had to be mapped bit by bit in a common effort of international division of laboratory labour.^19^ In 1964, the first International Histocompatibility Workshop kicked off a series of meetings that aimed at detecting and characterising HLA loci and defining antigens. Eventually, a worldwide standard of HLA nomenclature, typing sera, and typing techniques was established. Even if this definitory work was dominated by some twenty laboratories from a handful of countries, many more contributed by obtaining and providing different typing sera, the necessary reagents, including laboratories from the Soviet bloc.

As the contours of the HLA system and the distribution of its variants among populations began to emerge, it was estimated that a pool of at least several hundred potential recipients would be needed to find a good match for a cadaveric kidney.^20^ This meant that the patient pool of a single hospital or even a single city would be way too small. Therefore, transplantation pioneers started to recombine their growing patient pools into larger entities. In the wake of the third Histocompatibility Workshop, held in Italy in 1967, one protagonist of early immunogenetics, Dutch haematologist Johannes van Rood (1926–2017), launched the first European exchange organisation. The Eurotransplant foundation was meant to be a registry of potential recipients to optimise HLA matching. It was housed at the blood bank of Leiden University Hospital, which was the reference laboratory and co-ordination centre for transplantation in the Netherlands.^21^ Transplantation centres from the Netherlands, Belgium, West Germany, and Austria joined (as did Luxemburg in 1982) to exchange patient data and organs. Following this example, Scandiatransplant – for the Nordic countries – and France-Transplant were founded in 1969. In the UK, the National Organ Matching and Distribution Service was established in 1972, as was the North Italy Transplant Program.^22^

What did the exchange programmes do? Basically, they assembled, stored, and updated immunological and clinical information on potential kidney recipients, i.e. patients who were on chronic dialysis but otherwise healthy enough to undergo surgery at any moment. Transnational networks like Eurotransplant received this data input from the national waiting lists. The second basic function of the programmes was to act quickly when the critical moment arrived: when fresh kidneys were reported, the co-ordination centre would start the recipient selection procedure, which, from the 1970s, was not carried out by human decision makers but by computer algorithms. The model was the famous Eurotransplant computer programme, promising fair selection based on medical possibilities and urgency alone. If no matching recipient could be found in their own database, the organ would be reported to interconnected exchange programmes, e.g. from Eurotransplant to Scandiatransplant. To be sure, the choices that fed into the algorithm were man-made and represented another task in the portfolio of the exchange organisations: the background work of standardisation and quality control. What were the criteria for the acceptance of a patient into the waiting list? How was medical urgency defined? Which tissue incompatibilities should be tolerated? Documentation and research were constant activities, especially for Eurotransplant: defining typing techniques and reagents, assembling and evaluating data on the outcomes of transplantation, and making recommendations for treatment based on such evaluations.^23^

Here, Eurotransplant overlapped with the major professional association in the field, the European Dialysis and Transplant Association (EDTA, from 1981 EDTA–ERA (European Renal Association), later ERA–EDTA, now ERA). Its most important output were annual statistical surveys on dialysis and transplantation activity in centres which were willing to share their data. Eventually, Eurotransplant and Intertransplant would streamline their data gathering with the annual EDTA reporting.^24^ The EDTA Registry displayed the joint progress in the field of kidney replacement therapy, but it also ranked countries according to their performance in graft and patient survival rates and the ratio of dialyses and transplantations per million inhabitants – a striking example of ‘competition *through* co-operation’.^25^ East German physicians were eager to participate from early on – Dutz, Mebel, and Klinkmann were among the first socialist members – and during the 1970s, centres from all over Europe and from non-European countries bordering the Mediterranean contributed to what became the WHO reference statistics in the field of ESRD treatment.^26^

Geographical outreach that went beyond formal membership similarly characterised Eurotransplant statistics, which knew three categories of exchange partners: the core of participating centres in the Netherlands, Belgium, Austria, the FRG, and Luxemburg; sister organisations in Switzerland, the Scandinavian countries, France, and the UK; and a wider circle of ‘others’ in Europe and beyond, namely Italy, the GDR, the USA (from 1976), Iran (1977/8), Israel, the USSR, Czechoslovakia (from 1977), Turkey (from 1978), Spain, Greece, Poland (from 1979), Yugoslavia, Hungary (from 1980), Kuwait, Canada, Portugal (from 1981), and Saudi Arabia (from 1984).^27^ Interestingly, individual state-socialist countries appear in Eurotransplant reporting, but its socialist counterpart, Intertransplant, is never mentioned. In 1984, there were still no official connections between the two.^28^ This raises the question of what Intertransplant looked like from the inside.

## The making of Intertransplant: an East German perspective

The Intertransplant scheme was created as an adaptation of the multilateral Eurotransplant model to state socialism. Therefore, unlike Eurotransplant, Intertransplant was not a civil society initiative but evolved as a result of central planning. While Eurotransplant was realised through contracts between the Dutch foundation and individual members – the dialysis and transplantation centres – Intertransplant was a treaty between states, represented by the respective ministries of health care. It was signed by Bulgaria, Czechoslovakia, the GDR, Hungary, Poland, and the USSR in November 1980, joined in the following years by the non-European members Cuba, Mongolia, and Vietnam, and dissolved on 1 January 1991.^29^

However, the process of establishing Intertransplant had begun a decade before. As early as 1968, when ‘first’ transplantations had been carried out in several countries or were underway,^30^ the emerging state-socialist transplantation community started a conversation about joining their efforts. In 1969, nephrologists and urologists from the GDR, Hungary, Bulgaria, Poland, and the Soviet Union requested that their ministries of health care put the topic on the agenda of the regular conferences of socialist health ministers; they proposed installing a transnational expert group that would suggest a framework for collaborating on transplantation.^31^ From 1970/1, the name ‘Intertransplant’ was used.^32^ In parallel, bilateral organ exchange began to materialise between socialist states on the basis of Friendship Agreements, which allowed physicians, scientists, and technicians to visit health care facilities in the partner country and medical associations to organise joint conferences. GDR transplantologists also hoped to establish formal exchange ties with Sweden through the support of Nils Alwall, but they did not materialise.^33^ In 1971, East Germany shipped the first two organs abroad. No matching recipients had been found for the kidneys of an accident victim in Berlin, so they were transferred to Prague and transplanted to two Czech patients. In an interview, Moritz Mebel referred to the ‘“Intertransplant” organisation’ enabling this life-saving mission.^34^ Even if an Intertransplant spirit was undoubtedly already there, it seems that it was bilateral relations that initially facilitated this transnationalism in socialist kidney transplantation.

Even if Czech-German affiliations in medicine went back for decades, it is nonetheless remarkable that it was this duo which started the actual organ exchange. After the military suppression of the Prague Spring, which the GDR leadership fully supported, attitudes towards East German dialysis physicians had been rather hostile in Prague, to the extent that artificial kidneys produced in the GDR were put out of service.^35^ Following the hint of a Polish colleague, an immunologist from the East German reference laboratory refrained from going to Eurotransplant headquarters for training, ‘as a rejection on [Johannes van Rood’s] part may be expected in connection with the events in the ČSSR’.^36^ Repercussions had also been felt in international organisations, regarding East Germany’s ambition to host the annual conference of the EDTA and to be accepted into the ISN. In 1968/9, the Czech member of the EDTA council, nephrologist Albert Válek (1925–1995), stated that he would never agree to the GDR being nominated as a conference venue, as did several members from Western countries.^37^ Later in 1969, Válek came around, and thanks to Nils Alwall’s intervention, the GDR was nominated as a host for the EDTA conference of 1971 and joined the ISN.^38^ But at a conference held in West Berlin, Horst Klinkmann learned that Czechoslovak colleagues ‘did not adhere to the common stipulations of the CMEA [Council for Mutual Economic Assistance] countries with regard to the organisation of organ transplants, but went their own way’, oriented towards ‘the FRG and Austria in particular’.^39^ In kidney replacement therapy, relations with the Czech side only fully ‘normalised’ in the mid-1970s.^40^

This was when the East German Ministry of Health Care concluded new bilateral agreements with their counterparts in the ČSSR and Hungary, which specifically dealt with the exchange of donor organs. Objects of the agreement would later on be addressed in the Intertransplant treaty as well: shared clinical, immunological and surgical standards, and the regular exchange of waiting lists and of clinical and experimental experiences.^41^ Connections including occasional organ exchange also existed with Poland in the person of Tadeusz Orłowski (1917–2008) from the First Medical University Clinic in Warsaw, who in 1975 became head of the Transplantation Institute of the Medical Academy.^42^ Relations with the Soviet Union were less developed, despite official rhetoric to the contrary. In the early 1970s, one informer told his secret police officer that co-operation in kidney transplantation with the Soviet Union was non-existent.^43^ Only the news-breaking kidney flown in from Moscow in 1973 seems to have been a starting point for a good working relationship between the Friedrichshain transplantation centre and the Research Institute of Transplantology and Artificial Organs headed by Valery Ivanovich Shumakov (1931–2008).^44^

After the Intertransplant project had been jumpstarted through interconnected bilateralism in Central Eastern Europe by the middle of the decade, the first steps towards a multilateral solution were taken in 1975. A Standing Committee on Co-operation in Health Care was established within the CMEA. The first plan for the period of 1976 to 1980 defined eleven subjects or ‘complex problems’ of scientific collaboration in medicine. Subject no. 8 was devoted to ‘transplantations of organs and tissues and questions of transplantation immunology’. It was divided into several subfields, including kidney transplantation, organ conservation, artificial organs, and, last but not least, the establishment and consolidation of Intertransplant.^45^ The plan defined responsibilities for each subfield and more circumscribed contributions that institutions had to make according to their expertise. For the GDR, Moritz Mebel co-ordinated kidney transplantation and Horst Klinkmann artificial organs. The Berlin District Institute for Blood Donation and Transfusions was supposed to support the immunological work of their Czech colleagues.^46^

From the start, the national transplantation systems were represented by high-profile institutions and their heads, which ensured both the principle of hierarchy and economic organisation with clear responsibilities. Also, with limited resources for traveling to hard currency countries, this structure made sure that the best-connected people would bring the knowledge they picked up in the West back into the Intertransplant community. A case in point was the histocompatibility workshops Intertransplant members conducted, devised as preparation for the International Histocompatibility Workshops.^47^ For the GDR, the Friedrichshain transplantation centre and Moritz Mebel were Intertransplant liaisons, as were the National Institute of Haematology and Blood Transfusion in Budapest, the Transplantation Institute of the Warsaw Medical Academy, the Centre for Nephrology and Urology of the Medical Academy, Sofia, Shumakov’s Institute in Moscow, and the Institute for Clinical and Experimental Medicine (IKEM) in Prague, for their respective countries.

The IKEM soon assumed an outstanding position among the peer institutions. Within the CMEA Standing Committee on Co-operation in Health Care, political decisions were taken in Moscow, and the official language of complex problem no. 8 was Russian,^48^ but executive responsibilities were concentrated in Prague. The IKEM was a non-university institution founded in 1971 which reported directly to the ministry of health. Its Research Department for Organ Transplantation ran the major transplantation centre and the tissue typing reference laboratory in the ČSSR. From 1976, the IKEM computing centre operated a joint Intertransplant waiting list, and an IKEM-led working group evaluated the outcomes of transplantation and immunosuppressive therapy.^49^ In 1978, the IKEM group also co-ordinated the process of devising a more permanent structure for Intertransplant and drafted the ‘manual’, a guideline for the procedures of managing a joint waiting list and of exchanging organs, including criteria and standards for all aspects of the transplantation process. The Intertransplant treaty designated the IKEM as the headquarters, and, in 1981, distinguished IKEM transplantation surgeon Vladimír Kočandrle (1933–2017) was elected first president of the Intertransplant council.^50^

The pronounced role of Czech transplantation medicine does not mean that Czech actors simply had their way. In 1979, there were three competing proposals for an Intertransplant structure: one by the Czech delegation; another one by the CMEA, which had incorporated suggestions from the participating countries, and a last-minute one from the Soviet delegation. Moritz Mebel complained about the Soviet head of the secretariat, who ‘did not always demonstrate great expertise and sometimes left much to be desired in terms of form’.^51^ During the decisive meeting in Moscow, the delegations voted for the Czech proposal as a starting point for discussion, but it was difficult to resolve the critical issues.^52^ To begin with, the Czechs and Hungarians wanted the exchange programme to be a formal organisation. Instead, the Soviets ensured that Intertransplant became a legal agreement. A point that concerned the East German Ministry of Health Care was that member countries were not obliged to report all of their donor organs. ‘Optimal recipients’ in a shared waiting list would not be served preferentially, instead, member states could still prioritise less-than-optimal patients on the domestic waiting list.

Data gathering was supposed to serve at least two different purposes, drawing on both the annual EDTA statistical reports^53^ and the Eurotransplant waiting list. First, a cumulative patient database would allow a comparison of ‘the results and the quality of dialysis treatment in the Intertransplant member states’, and East German nephrologists expected to compare favourably.^54^ The same competitive perspective would be applied to transplantation performance. East German physicians and health bureaucrats frequently referred to Intertransplant statistics as provided by the IKEM just like they referred to EDTA statistics, to negotiate what was necessary, desirable, and possible for ESRD treatment in their country. Second, the shared data would also form an Intertransplant waiting list for kidney transplantation. A computerised selection mechanism was to ensure the most beneficial and effective matches between donor organs and recipients. Drawing on experiences with the EDTA statistics and for lack of domestic computing resources, East German physicians opted for a liberal exchange of (encoded) patient data in order to gain knowledge about the performance of East German kidney replacement therapy – the Czech colleagues at the IKEM had an efficient computer at their disposal. It was out of the question, though, that ‘persons whose data require special protection’ would appear in this database: patients treated at the Government Hospital. Sensitive health information on high-ranking party members and state officials was not to be disclosed to the socialist brother countries.^55^

After the Intertransplant treaty had been signed, hopes were that ‘in the nearest future a broader collaboration between’ transplantation programmes throughout the world would materialise.^56^ However, although Intertransplant was one of the rare instances where the Standing Committee enabled practical – not only scientific – exchange, complex problem no. 8 did not meet expectations. Transplantation and organ exchange characteristically relied on decentralised and flexible decision-making by heterogenous actors on local and national levels. Intertransplant was thwarted by rigid hierarchies and centralism, reporting duties, planning bureaucratism, and relative isolation from international developments, as former GDR health official Konstantin Rimkeit put it. The most productive part remained bilateral research co-operation between individual institutions.^57^

## Kidney exchange activity in the GDR

Given the European paradigm of HLA matches and the GDR’s all-in-all successful establishment of a domestic transplantation system, the question arises: what significance did transnational organ exchange have, in quantitative as well as qualitative terms? Statistics provided by the Ministry of Health Care may help here. Though we cannot be sure that the figures are entirely correct, they probably convey a fair impression of the quantitative dimensions.

Significant and continuous exchange both within the Intertransplant group and with Eurotransplant started in 1976/7.^58^ Exchange activity peaked in 1979, with seventy grafts going out and twenty-four coming in. Most kidneys were shipped to socialist countries, also in 1978/9 (and 1989), even before the Intertransplant treaty was negotiated and came into effect. Most kidneys were received from socialist countries between 1982 and 1984; after that, the numbers dropped to an insignificant level. This possibly reflects the market launch of cyclosporine A in 1983. With the specific suppression of the rejection response, matching became less crucial, and kidneys were generally transplanted more often in the donor region, as now a higher degree of HLA incompatibility could be tolerated. Thus, fewer organs were reported to the international exchange organisations.^59^ Exchange was still indispensable for the group of highly immunised patients who showed incompatibility with the bulk of the standardised typing panel and would run a huge risk of severe rejection.^60^ If we look at the number of kidneys transplanted within Intertransplant in the 1980s, the GDR provided 104 kidneys to Czechoslovakia, seventy-eight to Hungary, twenty to Poland, and thirteen to the Soviet Union. The other way around, seventeen grafts from Czechoslovakia were transplanted in the GDR, eight from Hungary, six from the USSR, and three from Eurotransplant (none from others).^61^

A more significant exchange with Eurotransplant from 1977 coincided with selected staff members of the Friedrichshain transplantation centre attending the annual Eurotransplant conferences and training workshops, as did scientists of the District Institute for Blood Donation and Transfusions and the Charité transplantation centre in the 1980s.^62^ Organ exchange with Eurotransplant continued until the autumn of 1989, albeit with considerable fluctuation. We have only scattered knowledge from some of the *Eurotransplant Annual Reports* about the destinations and origins of kidneys. According to the 1979 report, for instance, two kidneys from the GDR were transplanted in Italy and one in Turkey, while East German patients received two kidneys from West Germany, three from Dutch donors, and another kidney came from the Scandiatransplant network.^63^

From a different official source, we know that between 1967, when the transplantation programme started, and the end of 1988, 2,216 kidneys from cadaveric donors were transplanted in the GDR altogether.^64^ According to Table 1, only sixty-six – or three per cent – out of these 2,216 transplanted kidneys came from organised transnational exchange. As much as the sixty-six kidneys were beneficial to individual patients in the GDR, their sheer number seems not to have been essential for the functioning of the East German transplantation system. Moreover, only some of the kidneys reported or even of those received from abroad could actually be transplanted. Of the 35 kidneys received from Eurotransplant, only a little more than fifty per cent could be used for transplantation. The ratio was even worse for kidneys received from Intertransplant: only forty-nine out of 128 kidneys were transplanted to East German citizens. But the same was true the other way around. As far as the incomplete data allow us to conclude, from seventy-seven East German donors, or 144 potential kidneys, reported to Eurotransplant, only fifty-one transplantations resulted.^65^ Transplantation practice on both sides of the Iron Curtain meant that transnationally reported organs often were of minor quality (for various reasons) or had been taken from donors with rare blood group and tissue profiles, while ‘good’ grafts with ‘easy’ matches would be used on the spot.^66^

**Table 1.** Kidneys sent abroad/received from abroad and transplanted from 1971 to 1988

	Sent abroad			Received from abroad, x = transplanted					
	Inter	Euro	Total	Inter	x	Euro	x	Total	x
1971	2	0	**2**	0	0	0	0	**0**	**0**
1972	5	0	**5**	2	2	0	0	**2**	**2**
1973	0	0	**0**	0	0	0	0	**0**	**0**
1974	5	0	**5**	0	0	0	0	**0**	**0**
1975	4	0	**4**	4	1	0	0	**4**	**1**
1976	17	1	**18**	0	0	0	0	**0**	**0**
1977	20	16	**36**	15	2	8	5	**23**	**7**
1978	49	17	**66**	5	2	1	1	**6**	**3**
1979	49	21	**70**	10	5	14	6	**24**	**11**
1980	32	13	**45**	6	1	5	2	**11**	**3**
1981	20	6	**26**	7	5	5	3	**12**	**8**
1982	38	11	**49**	17	9	1	0	**18**	**9**
1983	33	17	**50**	29	6	0	0	**29**	**6**
1984	42	5	**47**	20	10	1	0	**21**	**10**
1985	24	4	**28**	4	3	0	0	**4**	**3**
1986	26	8	**34**	2	0	0	0	**2**	**-**
1987	23	14	**37**	6	2	0	0	**6**	**2**
1988	34	13	**47**	1	1	0	0	**1**	**1**
1989	50	14	**64**	4	2	0	0	**4**	**2**
**Total**	**473**	**160**	**313**	**132**	**51**	**35**	**17**	**167**	**68**

Note: Inter = Intertransplant; Euro = Eurotransplant

Source: In das Ausland verschickte/aus dem Ausland erhaltene u. transplantierte Nieren von 1971–31.12.1988, BArch DQ 1/14219. Figures for 1989 from Mebel, May and Althaus, *op. cit.* (note 3), 77, table 6. Other sources suggest slightly different numbers. Cf. Eurotransplant Foundation, *Eurotransplant Annual Report 1978*, table VIb; Aantal niertransplantaties centrum GEBPT (voorheen East Berlin) (received from the Eurotransplant public relations office via email on 17 June 2022).

We can also learn from Table 1 that the GDR was a net supplier of kidneys in trans-border exchange, shipping almost 633 kidneys to both Intertransplant and Eurotransplant while receiving only 167. Disadvantageous imbalances of this kind were no exception. Structural disproportions of giving and taking in kidney exchange were a constant irritation within the Eurotransplant community, for instance.^67^ They evolved because the treatment, despite its transnational quality, was informed by national differences in organising health care. These concerns included funding, reimbursement, and care infrastructure, but also bioethical debates and legislation.

The GDR had introduced legal regulation of organ transplantation in 1975, comparatively early in international comparison. Tailored towards the needs of transplantation medicine, it confirmed the practiced principle of presumed consent: deceased or brain-dead persons automatically became organ donors unless it was known that they had objected during their lifetime. From the start, East German ESRD specialists had thought it perfectly legitimate to use organs of a deceased person to save someone else’s life. The regulation of 1975 mirrored their ethical priorities, as it was rather laconic on cadaveric donors but rather detailed with regard to physical risks and emotional stress for living donors. It also obliged physicians to obtain and document graft recipients’ informed consent.^68^

Though socialist countries tended to dispose rather liberally of the dead bodies and body parts of their citizens,^69^ political systems did not correspond neatly with presumed versus informed consent approaches. Throughout the West, legal regulations and practices of cadaveric organ donation remained diverse and fluid. From the late 1970s, several West European countries enacted opt-out legislation (e.g. Spain in 1979, Austria in 1982, and Belgium in 1986).^70^ Significant differences, however, can be discerned regarding the measures taken to ensure informed decision-making. The GDR practised what is nowadays called a ‘hard opt-out’: relatives of cadaveric donors were neither asked for consent nor informed. Furthermore, a deliberate lack of transparency characterised its organ donation legislation. The Ministry of State Security had advised that the 1975 regulation be kept secret, as the opt-out paragraph could raise critical responses within the GDR and abroad, including the expression of ‘religious and also ethical-moral views’.^71^ While it was published in the Law Gazette, nonetheless, there was no information campaign that would have made citizens aware of it, let alone a public debate. Nor did the health administration create an official mechanism to register objection to being an organ donor.^72^

However, resolute pro-organ-harvesting legislation alone did not guarantee a satisfactory degree of implanting activity, as other variables left much to be desired. Especially in the 1980s, shortages, friction, and delays frequently disrupted the East German system of kidney transplantation. Insufficient conservation technology put the selection and preparation of patients on a tight time schedule. As dialysis treatment capacities and equipment were never sufficient, the patients’ state of health deteriorated, so they could not undergo transplantation. The paperwork necessary for keeping patient information up to date was overwhelming when personnel were already overburdened by clinical routines. Dialysis centres did not communicate properly with the co-ordinating office at the Friedrichshain transplantation centre, so patients flagged ‘ready for transplantation’ in the database often turned out not to be when a suitable organ became available. Also, immunological typing was delayed, and often, local brain death commissions could not be summoned in time. As a consequence, many organs had to be reported to the exchange organisations.^73^

Kidneys travelling thousands of miles to save lives remained solitary events that made the news but did not reflect clinical routines. In quantitative terms, over a stretch of almost twenty years, the GDR transplantation centres did not tangibly profit from international organ exchange. But does that mean this engagement was inefficient or even a failure? Archival sources indicate that medical professionals, as well as health bureaucrats, seemed to think the commitment was worth it. Then how did the GDR benefit from non-commercial organ exports if not by getting a fair quantity of organs in exchange?

## A network of exchange networks

Organ transplantation systems have been described as ‘networks heterogeneously recombining other networks’, such as energy, traffic, and telecommunication infrastructure.^74^ In fact, traffic flow and information management processes were critical preconditions of transplantation becoming a routine. But this observation can throw some light if applied on a different plane as well. Maybe participating in networks of organ exchange, even if not *per se* advantageous for the GDR, helped to successfully pursue other goals: proving capability, staying connected, being included, gaining recognition as a state, and having a foot in the door of international biomedical progress. Organ exchange networks were intertwined and intersected with other networks relevant to kidney replacement therapy. Besides organs and typing sera, knowledge, data, technical devices, and drugs circulated in this wider network of interconnected exchange relations, where giving and receiving did not necessarily come in the same currency. A negative balance in the shipping of organs could be economic when it indirectly helped to secure a positive balance in the flows of data, knowledge, or drugs.

Such an indirect exchange rationale had emerged already in the 1960s. At first, when GDR physicians still hoped to excel in kidney replacement treatment and sell homemade dialysis machines to the world, the lack of international recognition was in the foreground. Presenting at EDTA conferences, getting hold of offices, hosting an annual meeting, and the East German Association of Nephrology becoming a member of the ISN were seen as steps towards acceptance into the WHO, and more generally towards being a respected member of the international community, in terms of diplomatic relations with Western countries.^75^ International contact resulting from EDTA activity also helped to improve the emerging domestic system of ESRD therapy: Annual EDTA conferences and council meetings at alternating venues allowed GDR representatives to visit leading dialysis and transplantation centres and to arrange internships for their junior and senior physicians and their laboratory staff.^76^ Here, they could acquire hands-on knowledge of a variety of general and specific problems, e.g. tissue typing, performing kidney biopsies, how to use the respective new generation of artificial kidneys, how to manage preoperative blood transfusions and postoperative care, and how to run a transplantation centre. International contact directly improved the level of treatment through the transfer of knowledge,^77^ at least in the privileged top institutions.

In the 1970s, when the treatment system expanded but hopes to be among the top performers or at least to become self-reliant with regard to technology dwindled, the focus of exchange shifted towards a more material one. As dialysis technology, consumables, and immunosuppressant drugs had to be imported, commissioned testing of dialysis devices and membranes became a systemically relevant factor, as it saved hard currency and provided access to the latest technology.^78^ Horst Klinkmann’s excellent connections to the US and other parts of the world helped to establish the Rostock Medical University Clinic as the leading dialysis centre in the GDR, in his perception, actually the leading institution in the socialist bloc and the ‘acknowledged test clinic for all socialist countries.’^79^ What helped in this respect was that the East German Society of Nephrology, approved by the EDTA leadership, had assumed a co-ordinating function with regard to Eastern bloc members’ EDTA activities early on. Practically, this included getting people elected to office and securing conference venues in socialist countries. Klinkmann strategically rallied support among socialist as well as non-socialist colleagues to hold offices in international associations: in 1979–84, he served as president of the International Society for Artificial Organs, in 1975–8 and 1984–8 he was a member of the EDTA council, and in 1988 was elected its first and only president from a socialist country.^80^

Despite this very successful networking, from the mid-1970s, it became evident that the GDR would fall short of its self-proclaimed goals in kidney replacement therapy. There was a growing gap between the real achievements of an expanding transplantation system, international developments in the field, and the promises made to the population. The situation worsened in the 1980s, when a structural debt crisis put East German health care under enormous pressure. As the acquisition of hard currency through ‘immaterial export’ became an explicit task for university clinics, the testing of drugs for Western pharmaceutical companies intensified.^81^ This was especially the case for cyclosporine A, a drug manufactured by the Swiss Sandoz company, which dramatically increased graft and patient survival rates. Authorized in 1983; it soon became standard medication in countries with hard currencies. This made East German physicians and health officials worry, not only because it would make the GDR look bad abroad. The social contract of free, accessible and up-to-date health care was at stake, as East German patients and dialysis physicians learned about the new drug via mass media and West German relatives. A ‘massive ideological and moral pressure’ arose: ‘It is politically unacceptable that private individuals and institutions, including the church, conduct collections for Cyclosporine for our patients in Western countries or that kidney transplant patients illegally leave the GDR for the sake of Cyclosporine therapy’.^82^

With no financial means available for buying drugs on the market, a veritable campaign of clinical testing in the field of graft rejection therapy and prevention was launched. This also happened against the backdrop of other socialist countries like Bulgaria and Czechoslovakia successfully seeking co-operation with Western companies.^83^ The East German testing of graft protection medication was centralised at the Urological Clinic of the Charité University Hospital, in the hands of Moritz Mebel’s long-time co-worker Dietmar Scholz (b. 1942). Since the mid-1970s, he had been involved in the Charité administration, where he was in charge of negotiating clinical tests with Western pharmaceutical companies.^84^ In 1983, he was appointed consultant of the Ministry of Health Care for drug provision in the context of transplantation, which meant that every single dose given to GDR patients had to be approved and assigned by him. Scholz also toured cyclosporine symposia and other conferences around the globe, often paid for by drug manufacturers, to present results, negotiate further support, and acquire new contracts or participation in multicentric studies.^85^ In this vein, Eurotransplant workshops became platforms for meeting representatives of pharmaceutical companies so that the most urgently needed medications would be provided for free.^86^ From early 1984 until the end of 1985 alone, cyclosporine A, anti-thymocyte globulin (ATG), monoclonal antibodies, and medication countering cytomegalovirus infection, worth half a million *Deutsche Marks* (the West German currency), were imported via clinical testing.^87^

## Conclusions

If one would expect that reciprocal organ exchange was a prerequisite for a successful national transplantation system, the East German case can teach an interesting lesson. The GDR transplantation system was able to procure enough kidneys to keep the system running but failed to achieve the number of transplantations determined by the plan, which would have met the estimated needs of patients. However, to overcome the shortage, the priority was not importing but increasing the frequency and quality of domestic harvesting.^88^ Organs crossing the national borders were anything but instrumental for the system to work, though the insufficient number of organs increased inequalities between Berlin and the rest of the country, between normal citizens and privileged ones.^89^ Still, it made sense for the GDR to participate in organ exchange and even ship many organs abroad, as they were only one element in a wider network of exchange relations. What the GDR had to gain from networking and exchange in general was recognition: that it had a decent level of health care, that it could compete in specialised treatment, and moreover that it was capable of international co-operation and that it was a reliable partner. This reputation paid off most prominently when, in the 1980s, urgently needed medication could only be accessed through commissioned clinical testing. This was at a time when testing on humans had become more difficult in Western countries due to legal restrictions, reluctance on the part of patients, and general suspicion towards big pharma. GDR physicians and health officials were aware that they had something to offer to Western companies: a centralised, well-organised testing environment with clear-cut responsibilities, compliance with international standards of clinical testing, and no agitation to be expected from patients, relatives, or the critical public.^90^

Apart from these material benefits, being part of a data exchange community was equally important. In a country where sensitive statistical information like the rates of suicide, crime, and alcoholism were kept secret, it was desirable to have access to reliable data (and computing resources) via transnational networking. And *vice versa*: the EDTA Registry acknowledged that, due to the uniquely centralised data gathering process in the GDR, the East German questionnaire response rate was always a hundred per cent, and the data arrived in a handy single package.^91^ Figures from EDTA annual reports or similar international statistics circulated in the East German health care system, as they allowed performance and achievements to be evaluated ‘objectively’. Medical professionals frequently used them to underline their demands to the health administration. From the mid-1970s, they pointed out how the GDR, after a successful start in ESRD treatment, had managed to assert a leading position among the socialist countries (until it was ‘overtaken’ by Yugoslavia in the early 1980s) but fell behind in international rankings year after year. Though the absolute number of treatments continuously grew, the system stagnated and left East German physicians struggling to catch up with the Western average, at best.^92^

Despite many achievements realised self-reliantly or in joint socialist efforts, the GDR never escaped dependency on Western imports, be they material or immaterial. That does not mean the key point of this story is Western dominance – or Soviet dominance, either. Intertransplant provides an example of how Central Eastern European initiatives, invigorated by international connections, counterbalanced Soviet hegemony. At Intertransplant council and expert meetings, everybody would switch to English as soon as the official parts, conducted in Russian, had been finished. Even Moritz Mebel conceded that the obligation to communicate in Russian made co-operation more difficult.^93^ Though Mebel was a most loyal friend of the Soviet Union, his transplantation centre mostly worked with institutions in the neighbouring countries, Czechoslovakia and Poland. Geographical proximity and a long-standing shared academic tradition in Central Eastern Europe appear to have been more effective than the ideological agenda of Soviet leadership. The main actors at Intertransplant were Czechoslovakia, the GDR, and Hungary, while the Soviet colleagues and health administration kept to themselves. This is mirrored in Soviet kidney exchange activity, or lack of it. From 2,057 transplanted kidneys harvested in the USSR in the 1980s, only ten went abroad.^94^

To conclude, what did ‘therapies crossing borders’ mean in the case of East German kidney transplantation? From the perspective of organisational sociology, it has been argued that Eurotransplant lost its organ-supplying function soon after the arrival of cyclosporine. Monitoring, quality control, creating public trust, and overcoming the tenacious reluctance to donate organs moved to the fore, while organs kept being exchanged.^95^ Though critical discussion of organ donation was never the point in the GDR, a parallel can be seen in that organ exchange ceased to be (or maybe never was) primarily about organs. In the 1980s, it was, instead, about being part of an international professional and scientific community. Organ exchange, even on a very low level, rounded off a complex and shifting set of foreign relations in a particular field of medicine that helped stabilise the GDR healthcare system while also leaving it vulnerable at the same time. In kidney transplantation, as in politics more generally, the dynamics of opening up to the world and asserting independence were ridden with ambivalence and dialectical effects that were difficult to control.^96^

## Footnotes

1 ‘Dringliche Hilfe kam per Flugzeug aus Moskau’, *Berliner Zeitung*, 29, 317 (1973), 1.

2 ‘DDR half bei Vermittlung von Spenderniere’, *Neues Deutschland*, 32, 166 (1977), 2.

3 See Rosemarie Stein , Die Charité 1945–1992: Ein Mythos von innen (Berlin: Argon, 1992), 253–6.

4 See Jeannette Madarász-Lebenhagen , ‘Perceptions of Health after World War II: Heart Disease and Risk Factors in East and West Germany, 1945–75’, in Mary Fulbrook and Andre I. Port (eds), Becoming East German: Socialist Structures and Sensibilities after Hitler (New York: Berghahn, 2013), 121–40; Thorsten Halling , Friedrich Moll and Heiner Fangerau (eds), Urologie 1945–1990: Entwicklung und Vernetzung der Medizin in beiden deutschen Staaten (Berlin: Springer, 2015); Donna Harsch , ‘Gendering Health Politics: East and West German Healthcare Systems in Comparison, 1950–70’, in Karen Hagemann , Donna Harsch and Friederike Brühöfener (eds), Gendering Post-1945 German History: Entanglements (New York: Berghahn, 2019), 136–58; Christian Sammer , Gesunde Menschen machen: Die deutsch-deutsche Geschichte der Gesundheitsaufklärung, 1945–1967 (Berlin and Boston: De Gruyter-Oldenburg, 2020); Annette Hinz-Wessels , ‘Medizinische Verflechtung und Systemkonkurrenz im Kalten Krieg: Poliobekämpfung im geteilten Berlin’, Medizinhistorisches Journal, 55, 2 (2020), 132–71; Donna Harsch , ‘The Fight against Infant Mortality in Cold War Germany’, Journal of Modern History, 93, 2 (2021), 401–32.

5 On humanitarianism and medical aid, see, for example, Young-Sun Hong , Cold War Germany, the Third World, and the Global Humanitarian Regime (Cambridge: Cambridge University Press, 2015); Walter Bruchhausen and Iris Borowy , ‘Primary Health Care and Foreign Aid: A Tale of Two Germanys’, Journal of the History of Medicine and Allied Sciences, 79, 1 (2024), 65–89 37364178 10.1093/jhmas/jrad034.

6 See Johanna Folland , ‘“Not Even the Highest Wall Can Stop AIDS”: Expertise and Viral Politics at the German-German Border’, Central European History, 56, 2 (2023), 255–69; Volker Hess , ‘Beyond the Therapeutic Revolution: Psychopharmaceuticals Crossing the Berlin Wall’, in Mat Savelli and Sarah Marks (eds), Psychiatry in Communist Europe (Basingstoke: Palgrave Macmillan, 2015), 153–80.

7

Linda F. Hogle , Recovering the Nation’s Body: Cultural Memory, Medicine, and the Politics of Redemption (New Brunswick: Rutgers University Press, 1999), 96 . See also Günter Feuerstein , Das Transplantationssystem: Dynamik, Konflikte und ethisch-moralische Grenzgänge (Weinheim and Munich: Juventa, 1995), 16 .

8

Moritz Mebel , Gottfried May and Peter Althaus , *Der komplette Nierenersatz!?* Aufbau und Entwicklung der Nierentransplantation in der DDR (Lengerich: Pabst, 2003); G. Stein , B. Osten and H.J. Stolpe , ‘Möglichkeiten und Grenzen der Nephrologie in der ehemaligen DDR’, in W. Schulz and H. Brass (eds), Beiträge zur Geschichte der Nephrologie (Oberhaching: Dustri, 2017), 489–501 .

9 On ethical transgressions in organ donation, with a focus on heart and liver transplantation in the 1980s, see Stein, *op. cit.* (note 3), 114–5, 125–43, 253–70; Hogle, *op. cit.* (note 7), 84. A vague possibility of commercial traffic with East German donor organs is mentioned in Moritz Mebel, Einige Bemerkungen zu meiner Tätigkeit im Rahmen der Delegation des MHF unter Leitung des Staatssekretärs, Genossen Dr. Bernhard in Kuweit v. 30.11.–6.12.84, 7 December 1984, 3, Humboldt University Archive (hereafter abbreviated AHU), Med. Fak. 02, 038014/12 c.

10 Folland*, op. cit.* (note 6), 258, 268.

11 Seidel, Bericht über das 1. Kontakt-Gespräch mit OA Dr. Klinkmann, 28 February 1968, Stasi Records Office (hereafter abbreviated StUA), MfS, BV Rst AIM 2769/87, Vol. 1, sheet 79.

12 Mebel, May and Althaus, *op. cit.* (note 8), 90.

13 See Rainer Erices, Antje Gumz and Andreas Frewer, ‘Arzt, Akademiepräsident, Aufsichtsrat: Der DDR-Mediziner Horst Klinkmann im Dienst des Staates’, in Andreas Frewer and Rainer Erices (eds), *Medizinethik in der DDR: Moralische und menschenrechtliche Fragen im Gesundheitswesen* (Stuttgart: Steiner, 2015), 185–95.

14 Robert Weinberg (ed.), ‘A Life On The Left: Moritz Mebel’s Journey Through The Twentieth Century’, Marion J. Faber (trans.), *The Carl Beck Papers in Russian and East European Studies*, 1805 (2007), https://works.swarthmore.edu/cgi/viewcontent.cgi?article=1532&context=fac-history (retrieved 3 January 2025). Mebel’s estate in the Federal Archives was not yet accessible when this article was written.

15 During my research, Dutz’s correspondence, as preserved in his secretary’s files (hereafter abbreviated HDGZ), was temporarily deposited at the Berlin Institute for the History of Medicine and Ethics in Medicine. In the future, it will be transferred to the Humboldt University Archive.

16 Harald Dutz, Bericht über den III. Kongreß der European Dialysis and Transplant Association in Lyon vom 2.-4. Juni 1966 (EDTA), 23 July 1966, 2 and attachment, HGDZ; Analyse und Prognose der Problemkommission Nephrologie/Urologie, [1967], 7, Federal Archives (hereafter abbreviated BArch), DQ 109/266; Harald Dutz, Moritz Mebel and Klaus Precht , ‘Künstliche Niere – Nierentransplantation’, Das Deutsche Gesundheitswesen, 24, 39 (1969), 1831–44916104 .

17 Feuerstein, *op. cit.* (note 7), 15–16, 125.

18 In contrast with the European transplantation community, many US transplantologists doubted that HLA matching had any significance for transplantation outcomes. See Horst Klinkmann and Moritz Mebel, Reisebericht zur Reise vom 2.10. bis 1.11.1976 nach USA, 14 December 1976, 6, 9, 19–20, AHU, Med. Fak. 02, 038014/11; T.E. Starzl , S. Shapiro and L. Teperman , ‘The Point System in Organ Distribution’, Transplantation Proceedings, 21, 3 (1989), 3432–62662486 PMC2983103. For a critique of the politics of HLA see also Søren Bak-Jensen, ‘To Share or not to Share? Institutional Exchange of Cadaver Kidneys in Denmark’, *Medical History*, 52, 1 (2008), 23–46.

19

Erik Thorsby , ‘A Short History of HLA’, Tissue Antigens, 74, 2 (2009), 101–1619523022 10.1111/j.1399-0039.2009.01291.x.

20 Mebel, May and Althaus, *op. cit.* (note 8), 17.

21

Johannes Joseph van Rood , ‘A Proposal for International Cooperation in Organ Transplantation: Eurotransplant’, in E.S. Curtoni , P.L. Mattiuz and R.M. Tosi (eds), Histocompatibility Testing (Baltimore: Williams and Wilkins, 1967), 451–8.

22

Flemming Kissmeyer-Nielsen et al., ‘Scandiatransplant: Preliminary Report of a Kidney Exchange Program’, Transplantation Proceedings, 3, 2 (1971), 1019–294109156 ; J. Hors et al., ‘L’Association France-Transplant: I. *Organisation et fonctionnement*’, Nouvelle Presse médicale, 1, 19 (1972), 1269–724554688 ; S.D. Nelson and G.H. Tovey , ‘National Organ Matching and Distribution Service’, The British Medical Journal, 1, 5908 (1974), 622–410.1136/bmj.1.5908.622PMC16334244595177; Girolamo Sirchia et al., ‘The North Italy Transplant Program’s Organization, Policies and Activity: Problems and Proposals for the 1990’s’, Tissue Antigens, 34, 1 (1989), 78–83 2815058 10.1111/j.1399-0039.1989.tb01719.x.

23 Feuerstein, *op. cit.* (note 7), 125–6.

24 Bericht über die Teilnahme an der wissenschaftlichen Koordinierungsberatung von Spezialisten der Mitgliedsländer des Systems ‘Intertransplant’ zum ‘Projekt der Registrierung der Patienten im Dialyseprogramm’, Prag, 30./31.10.1984, 1, BArch, DQ 1/14218.

25 Folland, *op. cit.* (note 6), 258. On the EDTA and its Registry, see David N.S. Kerr , ‘The First Twenty-Five Years: EDTA to ERA’, Nephrology Dialysis Transplantation, 4 (1989), 411–1510.1093/oxfordjournals.ndt.a0919032505195; Anthony J. Wing et al., ‘Evolving Methodologies in Computerized European Registries’, Kidney International, 24, 4 (1983), 507–156358639 10.1038/ki.1983.187.

26

Nils Alwall , Harald Dutz and Horst Klinkmann , ‘A Survey of the Work of 35 Dialysis Centres in Eastern Europe and the Northern Countries over the Period 1946–July 1, 1964: Results and Further Planning of Interchange of Information’, Proceedings of the European Dialysis and Transplant Association, 2 (1965), 3–6 ; Hans Brynger et al., ‘Combined Report on Regular Dialysis and Transplantation in Europe, X, 1979’, Proceedings of the European Dialysis and Transplant Association, 17, (1980), 67017676, table II; Klaus Precht, Teilbericht – EDTA-Kongreß Paris 1981, 10 July 1981, 1, BArch, DQ 101/573; Klaus Precht, Teil I – Politischer Bericht des Besuchs des EDTA-Kongresses London, vom 19.-22. Juni 1983, 2, *idem*; Horst Klinkmann to Minister of Health Care Ludwig Mecklinger, 24 October 1984, 3, *idem.*

27 See Eurotransplant Foundation*, Annual Report 1976*, table VIa; *1977*, table V; *1978*, table Va; *1979*, table VIa; *1980*, table VIa; *1981*, tables VII and VIII; *1982*, tables V.4 and V.5; [*1983* missing]; *1984*, tables V.9 and V.10; *1985*, tables IV.10 and IV.8; *1986*, table 1.11; *1987*, table 1.11.

28 Bernard Cohen to Moritz Mebel, 12 September 1984, AHU, Med. Fak. 02, 038014/12 c; Mebel to Cohen, 4 October 1984, *idem*; Wolfgang Janisch, Reisebericht – Eurotransplant Meeting 1984 Leiden / NL vom 28.9. bis 2.10.1984, 3, 8, *idem.*

29 Abkommen über das internationale System der Zusammenarbeit auf dem Gebiet der Nierentransplantation ‘Intertransplant’, 11 December 1980, BArch, DQ 1/14218; Feuerstein, *op. cit.* (note 7), 124.

30 See Robert Langer (ed.), The History of the First Kidney Transplantation – a European Overview (Budapest: Semmelweis, 2019).

31 Harald Dutz to August Kaeding, 30 September 1968, HDGZ; Dutz to Prokop Málek, 14 October 1968, *idem*; Statement, 10 May 1969, *idem*; Dutz to Ministry of Health Care, 14 May 1969, *idem.*

32 Matthes, Organersatz, Transplantation, Immunologie, [January 1970], 3, BArch DQ 109/97; Harald Dutz, ‘Blutwäsche und Organersatz’, *Neues Deutschland*, 26, 251 (1971), 11; Maack/Klaus Richter, report, 6 December 1971, StUA, MfS, BV Berlin, AIM 1425/87, Vol. 1, sheet 60. As oral reports of informants were edited by secret police officers, I pair officers and informants as joint authors.

33 Harald Dutz, Bericht über die Councilsitzung der Europäischen Dialyse- und Transplantations-Gesellschaft (E.D.T.A.) am 17. und 18. Oktober 1969 in Barcelona, 27 October 1969, 4, BArch DQ 101/573.

34 ‘Nieren für passende Empfänger: Urologen sozialistischer Länder verstärken Zusammenarbeit’, *Neues Deutschland*, 27, 63 (1972), 4.

35 Seidel/IM ‘Ludwig’, Treffbericht, 15 November 1968, StUA, MfS, BV Rostock Nr. 698, AIM 2769/87, Vol. 3, pt 1, sheet 20–1; Thode/IM ‘Armin Hähnel’, Treffbericht, 19 November 1968, *idem*, sheet 24–5. For the background see Matthew Stibbe , ‘Ideological Offensive: The East German Leadership, the Prague Spring and the Warsaw Pact Invasion of August 1968’, in Kevin McDermott and Matthew Stibbe (eds), Eastern Europe in 1968: Responses to the Prague Spring and Warsaw Pact Invasion (Cham: Palgrave Macmillan, 2018), 97–123 .

36 Harald Dutz to Ministry of Health Care, 14 November 1968, HDGZ.

37 Seidel/IM ‘Ludwig’, *op. cit.* (note 35); Seidel/IM ‘Ludwig’, Treffbericht, 9 May 1969, MfS, BV Rostock Nr. 698, AIM 2769/87, Vol. 3, pt 1, sheet 34; Harald Dutz, Bericht über die Council-Sitzung der Europäischen Dialyse- und Transplantations-Gesellschaft (E.D.T.A.) in Paris vom 20.-23. März 1969, 21 April 1969, 2, HDGZ.

38 Seidel/IM ‘Ludwig’, Treffbericht, 9 May 1969, *ibid.*, sheet 33–4; [Horst Klinkmann], Reisebericht über kombinierte Studien- und Kongressreise nach Schweden vom 16.6.-13.7.1969. Teil I Erfüllung der politischen Aufgabenstellung, 16 July 1969, StUA, MfS, BV Rostock Nr. 698, AIM 2769/87, Vol. 3, pt 1, sheet 46.

39 Clasen/IM ‘Ludwig’, report, 27 November 1974, *ibid.*, sheet 151.

40 Clasen/IM ‘Ludwig’, report, 9 April 1975, *ibid.*, sheet 168. On post-1968 German-Czech relations see Matthew Stibbe , ‘Normalisation Across Borders: Official Cooperation and Contacts between East Germany and Czechoslovakia, 1969–1980’, in Kevin McDermott and Matthew Stibbe (eds), Czechoslovakia and Eastern Europe in the Era of Normalisation, 1969–1989 (Cham: Palgrave Macmillan, 2022), 259–89.

41 Entwurf Vereinbarung zwischen dem Ministerium für Gesundheitswesen der CSSR und dem Ministerium für Gesundheitswesen der DDR über den Austausch von Spenderorganen (Nieren) zur Transplantation, 15 November 1973, BArch, DQ 1/14218; Vereinbarung für die Zusammenarbeit zwischen der DDR und der UVR auf dem Gebiet der Nierentransplantation, 27 June/4 September 1975, DQ 1/12965.

42 Dutz to Tadeusz Orłowski, 8 October 1969, HDGZ; Dutz, Bericht über die Teilnahme am Symposium der Sektion Nephrologie von der Gesellschaft für Innere Medizin in Warschau (25.9.–2.10.1969), 9 October 1969, 1, *idem*; ‘Interflug beförderte lebensrettende Niere’, *Neues Deutschland*, 30, 9 (1975), 2.

43 Maack/Richter, *op. cit.* (note 32), sheet 60; Maack/Klaus Richter, Bericht, 17 April 1972, *idem*, sheet 63.

44 Mebel, May and Althaus, *op. cit.* (note 8), 93–4.

45

Konstantin Rimkeit and Fredo Wegmarshaus , ‘Internationale Beziehungen des Gesundheitswesens’, in Horst Spaar (ed.), Dokumentation zur Geschichte des Gesundheitswesens der DDR, Teil V (1971-1981) (Berlin: Trafo, 2002), 25–6; Moritz Mebel, Bericht über die Tagung zum Komplexproblem 8 ‘Transplantation von Organen und Gewebe und Fragen der Transplantationsimmunologie’ sowie zum Problem 8.4 ‘Organisation von Intertransplant’ des RGW in Prag vom 27. bis 28.3.1979, 19 April 1979, BArch, DQ 1/14218; Barleben, Protokoll über die Expertenberatung am 4. und 5.10.1979 im Sekretariat des RGW in Moskau zur Ausarbeitung des Abkommens über das System der Zusammenarbeit der RGW-Mitgliedsländer auf dem Gebiet der Nierentransplantation ‘Intertransplant’, *idem.*

46 Peter Althaus, Bericht Über die wissenschaftliche und koordinierende Beratung der RGW Mitgliedsländer zum Problemkomplex 8 – 0 ‘Transplantation von Organen und Geweben und Fragen der Transplantationsimmunologie’ in Budapest am 17.-18. März 1981, 28 March 1981, 6, BArch, DQ 1/14281; Maßnahmeplan der ständigen Kommission Gesundheitswesen 1985, Vorschläge zum Plan der Zusammenarbeit im Themenkomplex 8.0 des RGW-Problems 8.1.1. und 8.1.2. von 1986–1990, *idem.*

47

Gyözö Petrányi and R. Susan Hollán , ‘Joint Report from “HLA-DR Intertransplant Workshop 79”’, Tissue Antigens, 16, 1 (1980), 1–22 6162227 ; A. Májský and P. Kořínková, ‘VII. mezinárodní seminář Intertransplant o tkáňové typizaci (Leningrad, 18.–25. Listopadu 1981)’, *Časopis lékařů českých*, 121, 27 (1982), 862; Direktive für die Teilnahme der DDR-Delegation an der 8. Spezialistenberatung zum Problem 8.1. ‘Erarbeitung von Mitteln und Methoden zur Überwindung der Gewebeunverträglichkeit’ des Komplexproblems 8.0 ‘Organ- und Gewebetransplantation, Fragen der Transplantationsimmunologie’, 6 February 1985, 2–3, BArch, DQ 14218.

48 Mebel, *op. cit.* (note 45), 3; Althaus, *op. cit.* (note 46), 8.

49 Otto Schück (ed.), *IK+EM: Institute for Clinical and Experimental Medicine: Origins, Present, Future* (Prague: Avicenum, 1972); Mebel, May and Althaus, *op. cit.* (note 8), 95–6.

50 Althaus, *op. cit.* (note 46), 8.

51 Mebel, *op. cit.* (note 45), 3.

52 For this and the following see Barleben, *op. cit.* (note 45).

53 Data were to be retrieved using the questions from the EDTA questionnaires, see 30. Oktober 1984 Beratung im IKEM, Prag, Intertransplant, BArch, DQ 1/14218.

54 Bericht, *op. cit.* (note 24), 1.

55 Direktive, *op. cit.* (note 47), 3–4; Ulrich Schneidewind, Aktennotiz über ein Gespräch mit Genossen Prof. Dr. Klinkmann zum Vorschlag des Ministers für Gesundheitswesen der CSR, den Aufbau eines Registers für Dialysepatienten betreffend am 24.8.1984, BArch, DQ 14218; Edgar Harig and Ulrich Schneidewind, Aktennotiz über ein Gespräch mit Genossen Prof. Dr. Mebel am 11.9.1984, 1, *idem.*

56

E.M. Balakirev , ‘Scientific-Organizative Bases of the Collaboration in Renal Transplantation with the International System “Intertransplant”’, Scripta Scientifica Medica, 19 (1982), 100–2, 100.

57 Feuerstein, *op. cit.* (note 7), 16, 77, 121–2; Hogle, *op. cit.* (note 7), 103; Rimkeit and Wegmarshaus, *op. cit.* (note 45), 26–9; Konstantin Rimkeit and Fredo Wegmarshaus, ‘Internationale Beziehungen’, in Horst Spaar (ed.), *Dokumentation zur Geschichte des Gesundheitswesens der DDR, Teil VI* (Berlin: Trafo, 2003), 7–8. See also several plans and a contract on scientific co-operation between the IKEM’s Research Centre for Organ Transplantation, the Friedrichshain transplantation centre and the Charité’s research department for experimental organ transplantation for the periods 1982–5 and 1986–90, BArch, DQ 1/12567.

58 See also Mebel, May and Althaus, *op. cit.* (note 8), 53.

59 Janisch, *op. cit.* (note 28); Feuerstein, *op. cit.* (note 7), 306, 311–12. Delia Colombo and Enrico Ammirati , ‘Cyclosporine in Transplantation – a History of Converging Timelines’, Journal of Biological Regulators and Homeostatic Agents, 25, 4 (2011), 493–504 22217983 .

60

G.F. Hendriks et al., ‘Eurotransplant Experience with Highly Immunized Patients’, Scandinavian Journal of Urology and Nephrology Supplement, 92 (1985), 81–63914068 .

61 Mebel, May and Althaus, *op. cit.* (note 8), 98, table 8.1 [no source given, but consistent with annual surveys of kidney transplantation 1980–8, BArch, DQ 1/14219].

62 Janisch, Betr. Dienstreise in die Niederlande vom 19.–30.9.1977, AHU, Med. Fak. 02, 038014/11; Mebel to Lebentrau, 6 December 1979, AHU, Med. Fak. 02, 038014/12; May and Janisch, Betr. Studien- und Kongreßreise in die Niederlande 21.–27.9.81, Med. Fak. 02, 038014/12 a; Fünfhausen and Strauss, Stellungnahme zum Antrag auf Auslandsdienstreise für Frau Dr. sc. nat. Siegrid Leverenz nach Leiden und Amsterdam / Niederlande im September 1981, MfS BV Berlin, Abt. XX, Nr. 7982, sheet 37; Travel directive for Wolfgang Janisch, 3 September 1982, AHU, Med. Fak. 02, 038014/12 b; Janisch, *op. cit.* (note 28); Dietmar Scholz, Sofortbericht Eurotransplant meeting Leiden/Niederlande am 29.9.1984, 1 October 1984, StUA, MfS BV Berlin, Abt. XX, Nr. 12000, sheet 148–9; Dietmar Scholz, Sofortbericht Betr. Teilnahme am ‘Eurotransplant Summer Meeting’ in Lübeck-Travemünde vom 10.6.-15.6.1984, 19 June 1984, *idem*, sheet 152–3; Reiseantrag Dr. Groth zum Eurotransplant-Meeting, Leiden, September 1985, AHU, Med. Fak. 02, 038014/12 d; Mebel to Buhlert, 19 June 1987, AHU, Med. Fak. 02, 038014/12 e; Mebel to Thierfelder, 28 March 1988, *idem.*

63 Eurotransplant Foundation, *Eurotransplant Annual Report 1979*, 12, table VIa.

64 Nierentransplantationen in der DDR von 1967 bis 31.12.1988, BArch, DQ 1/14219.

65 Eurotransplant Foundation, *Eurotransplant Annual Report 1979*, table VIb; *1980*, table VIb; *1981*, table Va; *1982*, table V.5; *1984*, table V.10; *1985*, table IV.9.

66 See, e.g. Eurotransplant Foundation, *Eurotransplant Annual Report 1978*, 14; Feuerstein, *op. cit.* (note 7), 260–1, 306.

67 Feuerstein, *op. cit.* (note 7), 309–10.

68 ‘Verordnung über die Durchführung von Organtransplantationen vom 4. Juli 1975’, *Gesetzblatt der Deutschen Demokratischen Republik*, 1975, pt 1, 597-9.

69 See Daniela Koleva’s article in this special issue.

70 See WHO (ed.), *Legislative Responses to Organ Transplantation* (Dordrecht, Boston and London: Nijhoff, 1994).

71 Statement Oberst Filin, head of the MfS legal department, 20 January 1975, StUA, MfS, SdM no. 1015, sheet 148. See also Mielke to Mecklinger, 18 February 1975, *idem*, sheet 139.

72 Hogle, *op. cit.* (note 7), 68–9, 85; Jens Lohmeier and Dominik Gross , ‘Der Diskurs über die rechtliche Regelung der Organtransplantation in West- und Ostdeutschland (1960–1989): Eine vergleichende Studie’, Sudhoffs Archiv, 97, 1 (2013), 39–56.24195333

73 Moritz Mebel, Weiterentwicklung der Nierentransplantation in der DDR, December 1983, 5, BArch, DQ 1/12964; Hiller/IM ‘Dozent’, Zur Situation der Dialyse und Nierentransplantation in der DDR, 10 January 1985, StUA, MfS, AIM Nr. 10729/91, Vol. 2, sheet 184–6; [Lutz Trommer], Beratung zur weiteren Koordinierung von Nierenentnahmen sowie komplexer Organentnahmen für die Transplantation, 20 November 1986, 2, BArch, DQ 1/12965.

74

Ingo Braun and Bernward Joerges , How to Recombine Large Technical Systems: The Case of European Organ Transplantation (Berlin: Wissenschaftszentrum Berlin, 1993), 30 .

75 Rimkeit and Wegmarshaus, *op. cit.* (note 45), 24–35, 32–5; Rimkeit and Wegmarshaus, *op. cit.* (note 57), 5–16, 15.

76 See for example Dutz, *Bericht*, *op. cit.* (note 33), 4–5; Harald Dutz, Bericht über die Council-Sitzung der Europäischen Dialyse- und Transplantations-Gesellschaft (EDTA) am 18. April 1970 in Paris, 22 April 1970, 2–3, BArch DQ 101/573; Maack/Richter, *op. cit.* (note 32), sheet 60; Maack/Klaus Richter, Bericht, 22 February 1973, StUA, MfS, BV Berlin, AIM 1425/87, Vol. 1, sheet 67.

77 IM ‘Armin Hähnel’, Bericht, 20 June 1968, StUA, MfS, BV Rst, AIM 2769/87, Vol. 1, sheet 99; Information über Forschungsaufträge einer NSW-Firma für die Klinik für Innere Medizin der Wilhelm-Pieck-Universität, Rostock, 10 February 1982, *idem*, Vol. 2, sheet 181–93.

78 Interner Kontakt der Klinik für Innere Medizin der WPU Rostock zu Wissenschaftlern und Firmen aus dem NSW, 15/16 November 1982, *ibid.*, Vol. 2, sheet 243; Peltz, Bericht über einen Treff mit dem IMS ‘Ludwig’, 12 February 1985, StUA, MfS, BV Rostock Nr. 698, AIM 2769/87, Vol. 3, pt 3, sheet 448–9.

79 Seidel/IM ‘Ludwig’, Treffbericht, 31 October 1973, StUA, MfS, BV Rostock Nr. 698, AIM 2769/87, Vol. 3, pt 1, sheet 122–3; Clasen/IM ‘Ludwig’, report, 20 January 1976, *idem*, sheet 182; Wachlin/IM ‘Ludwig’, Tonbandabschrift Information über Dr. Bandel/BRD, 30 June 1978, *idem*, pt 2, sheet 350; Enka company to Horst Klinkmann, 11 January 1980, StUA, MfS, BV Rst, AIM 2769/87, Vol. 2, sheet 49–50.

80 Horst Klinkmann, Reisebericht 10.-14.3.1977 Helsinki, EDTA-Präsidiumstreffen, 14 April 1977, 1, 4, BArch DQ 101/573; Klaus Precht, Beratung der Vertreter der Gesellschaften für Nephrologie der sozialistischen Länder im Parkhotel Prag am 10. Juni 1980, 1–3, BArch DQ 101/573; Klaus Precht, Bericht, Teil I. Nationaler Kongreß für Nephrologie der DDR mit internationaler Beteiligung 15. bis 18. Mai 1983, Cottbus, StUA, MfS, AIM Nr. 10729/91, Vol. 2, sheet 131–2; Precht, London, *op. cit.* (note 26), 2–3; Klinkmann to Mecklinger, *op. cit.* (note 26); Klinkmann to Mecklinger, 9 July 1987, *idem.*

81

Rainer Erices , Andreas Frewer and Antje Gumz , ‘Testing Ground GDR: Western Pharmaceutical Firms Conducting Clinical Trials Behind the Iron Curtain’, Journal of Medical Ethics, 41, 7 (2015), 529–3325341732 10.1136/medethics-2013-101925; Volker Hess, Laura Hottenrott and Peter Steinkamp, *Testen im Osten: DDR-Arzneimittelstudien im Auftrag westlicher Pharmaindustrie, 1964–1990* (Berlin: be.bra, 2016). There was also material export, cf. Rainer Erices , ‘The East-West Blood Trade: How the German Democratic Republic Obtained Foreign Currency with Blood Products (1983–1990)’, Wiener Medizinische Wochenschrift, 168, 15/16 (2018), 384–9029992447 10.1007/s10354-018-0643-1.

82 Ciclosporin-Behandlung nach Nierentransplantation, [ca. 1984/5], BArch, DQ 1/12965. See also Dietmar Scholz to Zentraler Gutachterausschuss für den Arzneimittelverkehr, 21 March 1984, BArch, DQ 1/14474; Petzold, Aktennotiz zur Bereitstellung von Cyclosporin A, 3 April 1984, *idem*; Hiller/IM ‘Dozent’, *op. cit.* (note 73), sheet 183; Hiller/IM ‘Dozent’, Probleme in der Nephrologie, 5 August 1986, *idem*, sheet 281.

83 Precht, London, *op. cit.* (note 26), 1.

84 Dewitz, Vorschlag zur Registrierung eines GMS, 1 April 1987, StUA, BVfS Berlin, AGMS 4314/91, sheet 205–6; Harald Dutz, reference letter, 29 June 1979, HDGZ.

85 Protokoll der Beratung zum Sortiment an Immunsuppressiva für die Organ- und Knochenmarkstransplantation – Nachsitzung zur 246. ZGA-Sitzung am 26.1.1984, 20 March 1984, 6, BArch DQ 1/14474; Mebel, Symposium in Warschau, 16 May 1984, AHU, Med. Fak. 02, 038014/12 c; Scholz, Sofortbericht Dienstreise zum 2. Kongress der E.S.O.T. in München/BRD und Präkongress-Symposium Ciclosporin-Therapie nach Nierentransplantation vom 25.–30.11.1985, 1 December 1985, StUA, BVfS Berlin, AGMS 4314/91, sheet 86–7; Mebel to Thierfelder, 17 December 1985, AHU, Med. Fak. 02, 038014/12 d; Koroschetz/KP ‘Charité’, Bericht, 24 October 1986, StUA, BVfS Berlin, AGMS 4314/91, sheet 114–20; Travel application Moritz Mebel and Dietmar Scholz, AHU, Med. Fak. 02, 038014/9; Dewitz/GMS ‘Hans’, Bericht. Kommerzielle Kontakte mit NSW-Firmen, 23 July 1987, StUA, BVfS Berlin, AGMS 4314/91, sheet 235–6; Mebel to Thierfelder, 28 August 1987, AHU, Med. Fak. 02, 038014/12 e; Dewitz/GMS ‘Hans’, Bericht. Kontakte zur Firma ‘Bayro-Pharm’ (BRD, Tochtergesellschaft von Bayer), 5 July 1988, StUA, BVfS Berlin, AGMS 4314/91, sheet 272–4; Scholz, Kurzbericht über XII. Int. Congr. of the Transplantation Society Sydney, 14.–19.8.88, *idem*, sheet 287.

86 Scholz, Leiden, *op. cit.* (note 62), sheet 149; Scholz, Travemünde, *op. cit.*, (note 62), sheet 153.

87 Petzold to Möller, 12 October 1984; BArch DQ 1/14474, Probst, Arzneimittel für die Transplantationschirurgie, 6 December 1984, *idem*; Protokoll, *op. cit.* (note 85), 3; Mebel to Thierfelder, 17 December 1985, *op. cit.* (note 85); René Vonlanthen/Cilag AG to Mebel, 18 February 1986, AHU, Med. Fak. 02, 038014/12 d; Mebel to Thierfelder, 29 [May] 1986, *idem*; Mebel and Scholz to Großer, 24 September 1986, *idem*; Scholz, Reisebericht, 29 September 1986, *idem.*

88 Mebel, *op. cit.* (note 73); [Trommer], *op. cit.* (note 73), 1–2.

89 Hogle, *op. cit.* (note 7), 85; Stein, Osten and Stolpe, *op. cit.* (note 8), 489–500.

90 Klinkmann, Besuch des Forschungslaboratoriums der Firma Cordis Dow in Concord/Californien, [1978], StUA, MfS, BV Rostock Nr. 698, AIM 2769/87, Vol. 3, pt 2, sheet 346–7; Hintergründe und Zielstellungen kommerzieller Angebote des Schering-Konzerns an die DDR, die unter maßgeblicher Einflußnahme des erkannten DDR-Feindes […] stehen, 11 October 1985, StUA, BVfS Berlin, AGMS 4314/91, sheet 75; Dewitz/GMS ‘Hans’, Bericht. Medikamententestung in der DDR, 18 February 1988, *idem*, sheet 260. See also Hess, Hottenrott and Steinkamp, *op. cit.* (note 74), 179–83.

91 Anthony Wing to Harald Dutz, 29 June 1981, BArch, DQ 101/573; Precht, London, *op. cit.* (note 26), 2.

92 Wachlin/IM ‘Ludwig’, Mündl. Information zur Kapillarniere, 16 December 1977, StUA, MfS, BV Rostock Nr. 698, AIM 2769/87, Vol. 3, pt 2, sheet 265; Wachlin/IM ‘Ludwig’, *op. cit.* (note 11), sheet 280; Moritz Mebel to Ludwig Mecklinger, 26 April 1983, BArch DQ 1/12964; Mebel, *op. cit.* (note 73); Klinkmann to Mecklinger, *op. cit.* (note 26), 3; graphs and tables on haemodialysis in international comparison, [ca. 1984/5], StUA, MfS, AIM Nr. 10729/91, Vol. 2, sheet 270–3; Hiller/IM ‘Dozent’, *op. cit.* (note 82), sheet 276, 278–82, 285; Dewitz/GMS ‘Hans’, Bericht. Dr. […] (Innere Medizin, UG), 25 January 1989, StUA, BVfS Berlin AGMS 4314/91, sheet 299. See also Stein, Osten and Stolpe, *op. cit.* (note 8), 492.

93 Mebel, *op. cit.* (note 45), 3; Althaus, *op. cit.* (note 46), 8.

94 Mebel, May and Althaus, *op. cit.* (note 8), 98, table 8.1.

95 Feuerstein, *op. cit.* (note 7), 278–9, 311–13, 392, 394–8, 414.

96 Oliver Bange, ‘The GDR in the Era of Détente – Conflicting Perceptions and Strategies, 1965–1975’, in Poul Villaume and Odd Arne Westad (eds), *Perforating the Iron Curtain – European Détente, Transatlantic Relations, and the Cold War, 1965–1985* (Copenhagen: Museum Tusculanum Press, 2010), 57–77; Stibbe, *op. cit.* (note 40); Folland, *op. cit.* (note 6), 269.

